# Reflections on the Cost of "Low-Cost" Whole Genome Sequencing: Framing the Health Policy Debate

**DOI:** 10.1371/journal.pbio.1001699

**Published:** 2013-11-05

**Authors:** Timothy Caulfield, Jim Evans, Amy McGuire, Christopher McCabe, Tania Bubela, Robert Cook-Deegan, Jennifer Fishman, Stuart Hogarth, Fiona A. Miller, Vardit Ravitsky, Barbara Biesecker, Pascal Borry, Mildred K. Cho, June C. Carroll, Holly Etchegary, Yann Joly, Kazuto Kato, Sandra Soo-Jin Lee, Karen Rothenberg, Pamela Sankar, Michael J. Szego, Pilar Ossorio, Daryl Pullman, Francois Rousseau, Wendy J. Ungar, Brenda Wilson

**Affiliations:** 1 Health Law Institute, University of Alberta, Edmonton, Alberta, Canada; 2 Department of Genetics, UNC School of Medicine, Chapel Hill, North Carolina, United States of America; 3 Center for Medical Ethics and Health Policy at Baylor College of Medicine, Houston, Texas, United States of America; 4 Department of Emergency Medicine, University of Alberta, Edmonton, Alberta, Canada; 5 School of Public Health, University of Alberta, Edmonton, Alberta, Canada; 6 Institute for Genome Sciences & Policy and Sanford School of Public Policy, Duke University, Durham, North Carolina, United States of America; 7 Biomedical Ethics Unit, McGill University, Montreal, Quebec, Canada; 8 Department of Social Science, Health & Medicine, King's College London, London, England; 9 Institute of Health Policy, Management and Evaluation, University of Toronto, Toronto, Ontario, Canada; 10 Bioethics Programs at the Faculty of Medicine, Université de Montréal, Montreal, Quebec, Canada; 11 Genetic Counseling Program (Johns Hopkins University [JHU];National Human Genome Research Institute [NHGRI], Social and Behavioral Research Branch [SBRB];NHGRI/National Institutes of Health [NIH]), Bethesda, Maryland, United States of America; 12 Department of Public Health and Primary Care, KU Leuven, Leuven, Belgium; 13 Stanford Center for Biomedical Ethics, Stanford University, Stanford, California, United States of America; 14 Department of Family & Community Medicine, University of Toronto; Granovsky Gluskin Family Medicine Centre, Mount Sinai Hospital, Toronto, Ontario, Canada; 15 Faculty of Medicine, Memorial University of Newfoundland, St. John's, Newfoundland and Labrador, Canada; 16 Department of Human Genetics, Faculty of Medicine, McGill University, Montreal, Quebec, Canada; 17 Department of Biomedical Ethics and Public Policy, Graduate School of Medicine, Osaka University, Suita, Osaka, Japan; 18 Institute for Integrated Cell-Material Sciences, Kyoto University, Kyoto, Japan; 19 Program in Science, Technology, and Society, Stanford University, Stanford, California, United States of America; 20 National Human Genome Research Institute, National Institutes of Health, Bethesda, Maryland, United States of America; 21 Department of Medical Ethics and Health Policy, Perelman School of Medicine at the University of Pennsylvania, Philadelphia, Pennsylvania, United States of America; 22 Centre for Clinical Ethics (a joint venture between Providence Healthcare, St. Joseph's Health Centre, and St. Michael's Hospital), University of Toronto McLaughlin Centre and Joint Centre for Bioethics, Toronto, Ontario, Canada; 23 The Centre for Applied Genomics, Hospital for Sick Children, Toronto, Ontario, Canada; 24 University of Wisconsin Law School, Madison, Wisconsin, United States of America; 25 Morgridge Institute for Research, Madison, Wisconsin, United States of America; 26 CHU de Québec Research Center, Quebec City, Quebec, Canada; 27 Faculté de médicine, Université Laval, Quebec City, Quebec, Canada; 28 Program of Child Health Evaluative Sciences, Hospital for Sick Children, Toronto, Ontario, Canada; 29 Department of Epidemiology and Community Medicine, University of Ottawa, Ottawa, Ontario, Canada

## Abstract

The future clinical applications of whole genome sequencing come with speculation and enthusiasm but require careful consideration of the true system costs and health benefits of the clinical uses of this exciting technology.

## Summary

The cost of whole genome sequencing is dropping rapidly. There has been a great deal of enthusiasm about the potential for this technological advance to transform clinical care. Given the interest and significant investment in genomics, this seems an ideal time to consider what the evidence tells us about potential benefits and harms, particularly in the context of health care policy. The scale and pace of adoption of this powerful new technology should be driven by clinical need, clinical evidence, and a commitment to put patients at the centre of health care policy.

## Introduction

The upfront cost of sequencing an individual's entire genome is decreasing rapidly. As a result, whole genome sequencing (WGS) is becoming feasible for broad use in both research and clinical care. (In this article, by WGS we mean both WGS and other approaches, such as whole exome sequencing [WES] that, while not as comprehensive as WGS, nevertheless analyze a broad swath of the human genome.) Not surprisingly, this tremendous technological advance has resulted in a great deal of enthusiastic speculation about public uptake and clinical application. There is significant momentum around the idea of using WGS as a clinical tool in the near future [1]. Indeed, some institutions are already seeking to integrate WGS into their clinical programs [2]. The US press has even suggested that the drive for some institutions to develop the necessary technological capacity is akin to a genomics "arms race" [3],[4].

Given this interest and the concomitant investment in both genomic and clinical translational research, we should consider how best to frame health policy discussions about the utilization of these emerging sequencing technologies. For example, for many genomic funding agencies and some researchers, adopting WGS into routine clinical care is an explicit aspiration. Indeed, WGS has been called a revolutionizing diagnostic tool [5],[6] that will have a profound impact on the practice of medicine [7]. While inexpensive and efficient, WGS is an impressive technological achievement, with the potential to serve as the foundation for new approaches to screening, diagnosis, risk prediction, and prognostic platforms in clinical practice; the actual impact it will have on health and health care systems is far from certain.

In this article, we highlight policy issues that warrant thought regarding the applications/uses of WGS in clinical care and within health systems. As with any new technology, decisions about clinical use should, as much as possible, be based on the best available evidence and on consideration of potential benefits and harms [8]. History tells us that without careful consideration of the social forces that influence technological implementation and their public and social costs, a less than ideal utilization policy can emerge [9],[10]. As some seek to introduce WGS into clinical use—including what has been called a "genome-based assault on cancer" [4]—a detailed reflection on its clinical applications seems warranted. Indeed, as enthusiasm grows and speculation on a range of applications intensifies, the timing for this kind of policy analysis seems ideal.

Here we seek to highlight the most promising areas for the application of WGS, whilst considering areas where claims of its clinical and social utility may be overstated. We also consider, from a health policy perspective, how best to guide discussions about the implementation of this emerging technology.

## Public and Scientific Enthusiasm for WGS

Success stories of WGS abound in the popular press [11],[12]. Thousands of individuals currently have their genomes sequenced each year in the clinical, research and, to a lesser extent, direct-to-consumer context. And, in certain clinical situations, WGS helps to provide a more definitive diagnosis (e.g., in unusual and rare conditions that seem likely to have a genetic cause). For rare inherited conditions and some cancers, WGS has even led to improved medical management of patients [13]. Given these early successes, it is no surprise that there have been many enthusiastic predictions about the possible clinical value of WGS—particularly in the context of personalized medicine. One industry commentator, for example, has claimed that the rise in cancer rates "can be fixed" with genome sequencing and personalized medicine [14]. What impact might this type of discourse have on health policy?

Scientific and public enthusiasm for an emerging area is a common feature of the innovation process [15]. This enthusiasm and the associated public representations help to build institutional momentum and attract funding from both the public and private sectors [16]—a process that is particularly important for big, complex, and expensive areas of scientific inquiry, like genomics [17]. But research tells us that this kind of enthusiasm can, for better or worse, also impact how an area is represented, including framing the speculation about clinical utility and health benefit.

There is a growing literature on how a range of social forces and publication trends can lead to exaggerated claims of future clinical benefit [18]–[21]. It has, for example, been noted that positive "spin" exists in peer-reviewed articles [22], institutional press releases [23], and the popular press. Growing commercialization and translation pressures, the need to attract research support in a highly competitive funding environment, and the simple momentum caused by the commitment of a large number of researchers and resources [17] (also known as a "scientific bandwagon") [24] can distort public communication on this issue and thus public expectations [25]. These distortions, together with enthusiasm from funding entities, media coverage, and the positioning of WGS and personalized medicine as a tool for regional economic growth [26], may influence our thinking on how best to deploy WGS technologies within health care systems.

Health policy deliberations need to be aware of these forces and their impact on the representations and perceptions of the value and cost of high profile technology like WGS. Spectacular technological advances have led to the dramatic decrease in cost of sequencing [27], and this decrease is often treated as sufficient justification for its clinical application. A US$1,000 price tag does bring WGS data within reach for many. However, WGS brings with it more costs—both monetary and beyond—than the charges for sequencing. Upstream costs include creating and validating the institutional and technological infrastructure for both the production and storage of sequence data that follow clinical laboratory standards and for the interpretation and confirmation of WGS results. The latter can frequently be laborious, expensive, and highly time-consuming. This has led many to joke about of the US$1,000 genome and the US$1 million interpretation [28].

Moreover, the downstream costs of a diagnostic intervention can far outweigh the upfront costs of the initial test [29]. This is especially true for tests that generate a large amount of information, and potentially large amounts of ambiguous information as well as false positives and incidental findings. The downstream resource and health consequences of ambiguous results are substantial and can include clinical follow-up, additional tests, and also unnecessary surveillance and interventions—as is seen with other technologies, such as has happened, for example, with the introduction of prostate specific antigen (PSA) testing [9]. In clinical practice, there is rarely such a thing as a "low cost" test; the "low cost high value" WGS may be rarer still.

## Clinical Utilization of WGS

Lower cost sequencing has fostered the idea that there will be a high degree of both consumer and clinical utilization of WGS [3],[30], as captured by the suggestion that soon "everyone will be sequenced" [31]. There is little doubt that the application of WGS in the research setting is shedding new light on the molecular mechanisms that influence health, disease, and drug response. Also, there are significant social forces, particularly in the US and UK where this field is often cast as a potential engine of economic growth, driving its clinical implementation [4]. Nevertheless, we need to bear in mind that its uses in research do not necessarily imply equivalent utility in the clinic. Utility in a clinical setting depends on many—and very different—factors, and must take into account not only such performance characteristics as sensitivity, specificity, and positive and negative predictive value, but also demonstration of beneficial impact of using the test on patients' health, or on health services delivery. Failure to do so can trigger overt harm to patients in addition to excessive cost to the health care system [9].

It is clear that genomic sequencing will prove to be a useful diagnostic approach in specific situations [32]. For example, it will allow the identification of a causative mutation in patients with genetically heterogeneous disorders (in which mutations in many different genes can result in a similar phenotype), in children with complex unexplained co-morbidities, and in individuals with strong family histories of an enigmatic disorder. Although more work needs to be done to demonstrate clinical utility, promising opportunities exist in the realm of cancer treatment. For example, genome-scale sequencing of tumors may provide important information regarding the mutations that drive a patient's malignancy and so guide their treatment [33], with one of the potential beneficial by-products of WGS being drug dosing and pharmacogenomics applications.

In contrast to these successes, there are few data and little compelling support to suggest that WGS of individuals with common diseases will result in clinically actionable information, or that whatever benefits are accrued might outweigh the burdens of, for example, false positive results or the follow-up investigation of ambiguous results. Common diseases that, by definition, affect the greatest number of individuals, have a relatively low genetic component, placing an inherent ceiling on the usefulness of genomic information to meaningfully inform individuals regarding these disorders [34]. This in itself supports the adoption of a cautious, if not outright skeptical, perspective regarding the impact of WGS on the clinical management of common diseases and thus more modest expectations of a revolution in medical care, at least in the short term.

As mentioned above, there is a high risk of generating a lot of ambiguous information when a tremendously broad test such as WGS is used clinically. It is a well-supported tenet of medical practice that overly broad testing can cause considerable harm owing to the inevitable trade-off between sensitivity and specificity [35] requiring such testing to be carefully used. This caution regarding the use of non-specific testing has particular resonance when considering the application of WGS in healthy members of the population. In the public health setting, the probability that any specific variant is meaningful is low due to the rarity of disorders with a strong genetic cause in the general population.

While the balance of the clinical benefits and harms of WGS in otherwise healthy people may not currently support its adoption as a diagnostic tool, some communities outside of health care are already utilizing sequencing technology (via the private sector) to provide answers to questions that are not credibly available in any other manner, perhaps most notably in genealogy. The meaningfulness of WGS to these communities is difficult to refute. Advocates of WGS, and personalized medicine more generally, often promote the idea that more data is always better and that "knowledge is power" [36], and that genomics will inevitably empower patients and promote individual control over health (Box 1). The push to embrace WGS is inextricably linked to this vision of empowerment, particularly in the context of genetic risk information [37]. However, the provision of such information will create clinical challenges, including straining the physician/patient relationship by shifting more responsibility and expectations to the patient [38],[39]. More fundamentally, there is little evidence to support the basic premise implied by the empowerment rhetoric—namely that individuals will use genomic risk information to adopt a healthier lifestyle and, thus, reduce their risk for chronic diseases. In fact, existing research tells us that individuals do not alter their behaviour on the basis of genetic risk information [40]–[43]. Indeed, promoting meaningful behaviour change is tremendously difficult, particularly on a population level [44]. Hence, the value of WGS in this space—that is, in the context of empowerment—is conditional upon the development of effective behaviour change interventions.

**Box 1. The Rhetoric of Empowerment**

The ideas of empowerment and personal choice are significant aspects of the popular culture messaging around WGS, particularly in the context of personalized medicine. Below are a few examples of how this message is framed in various domains.

"The success of personalized medicine will come about only when we each take responsibility for our health. Health care providers can help, but they cannot drive your bus… [there are] things you can do now to take full advantage of the potential for personal empowerment. If you follow these recommendations, you will truly be on the leading edge of this new revolution" [48].

"WGS is not a panacea for all that ails humankind, but a powerful new tool that can catalyze our understanding of the genome and thereby empower patients" [49].

"Advances in genomic and molecular medicine hold the potential to radically transform human health by enabling much more precise prediction, prevention, and treatment of disease on an individual level… The Center's mission is to empower patients to understand their unique health needs…" [50].

"It [personalized medicine] is proactive and participatory, engaging patients in lifestyle choices and active health maintenance to compensate for genetic susceptibilities" [51].

"There will be a greater emphasis on the physician-patient relationship as we team together to develop more accurate and personalized care plans. Our ultimate goal is to empower our patients and our community towards greater health" [52].

In the context of utility it is also worth reflecting on the predictions of high uptake among the general population. Previous experience with high-throughput DNA technologies suggests the need for caution and an expectation that the utility of platform technologies such as WGS will be highly variable. Microarrays have played an important role in the diagnosis of developmental disorders, but their use in pharmacogenetics has thus far been clinically disappointing, in part due to an absence of evidence that they produce convincing outcomes. Array-based susceptibility testing for common diseases has also failed to garner clinical adoption, and where it has been commercialized as direct-to-consumer services there has been only modest uptake [45]. Despite claims that inexpensive WGS will lead to widespread use on a population level, there is little evidence, at this stage, to suggest that it will be widely adopted [46].

## Moving Forward

WGS holds undeniable promise as a diagnostic tool in certain clinical situations, and might also contribute to improving public health if used judiciously on an evidence-base basis [47]. However, its promise, coupled with the cautions noted above, argue for careful consideration as we seek to craft policy regarding its transition from research to clinical practice.

Characterizing the benefits and costs of specific applications of WGS will need to take full account of the upfront investment and downstream clinical practice implications. It will require the comparison of WGS-augmented care with current clinical practice and with care pathways that utilize alternative testing technologies.

Remembering that inefficient use of limited resources reduces the scale and quality of health care available for others, health systems will need to assess carefully the benefits of WGS that they wish to pay for and the quality of evidence they require to accept the benefits as demonstrated. Given the low unit cost of WGS, the risk of moving quickly from research and clinical practice may be substantial, and health systems will need to consider how to protect themselves from the costs of over-testing and the potential burden of false positives, in the absence of clear value criteria.

Clarifying the evidence hurdles facing WGS also will benefit the research community. By signaling clearly the type of evidence required to support a decision to provide funding to cover the costs of testing and related services, health systems will enable researchers and investors to prioritize alternative research investment opportunities to focus on those that have the greatest value.

## Conclusion

There are, of course, many other issues that need to be considered as WGS becomes more common, including concerns about genetic discrimination, issues of consent (e.g., to what degree should or could biological relatives be engaged in the consent process), and the direct-to-consumer provision of WGS. In addition, there are likely to be a range of translation issues, such as uncertainty about the role and impact of intellectual property (Box 2). Also, the diversity of health insurance systems and health economic policies in various countries will undoubtedly affect the way new technology is incorporated into clinical practices. But while these, and other, issues require further reflection, we already know enough to provide advice for the framing of health policy.

**Box 2. WGS and the Impact of Intellectual Property**

While intellectual property (IP) complexities may arise that concern WGS, they are unlikely, for a number of reasons, to come from gene-based patent claims [53],[54]. The policy rationale for exclusive rights in DNA-based diagnostics has historically been weak [55],[56]. And the recent decision by the Supreme Court of the United States, which declared that a naturally occurring DNA segment is a product of nature and therefore not patentable, will weaken patent related hurdles to WGS [57].

However, other forms of IP present challenges, such as data-hoarding practices in both academia and industry. Access to genomic data held in the private databases of both sectors is needed to advance science and to interpret diagnostic tests. Myriad Genetics' proprietary database, for example, is based on a million tests performed when Myriad's patent rights were presumed valid [58]. Lack of access to this data prevents the external validation of clinical interpretation, verification testing, and clinical research on BRCA gene mutations. Inaccessible data will also limit the comprehensiveness of core genomic databases, impoverishing the public domain. In response, innovative models are emerging at, for example, public research institutions to re-create public domain data resources where external validation is possible [59].

Translating new data into useful clinical information will require data-sharing, interoperability, and database infrastructure (and stable funding to ensure reliability, access, and curation). Interoperability includes legal regimes that accommodate differences in privacy laws and informed consent to enable the use of stored datasets. Patient groups are becoming increasingly active in establishing platforms through which patients and other individuals may contribute their own genetic and other health information [60]. Patients and consumers need to be at the table when decisions about pooling and sharing data are made. Widespread data sharing also entails risks to privacy. Therefore policies to promote data sharing will require a legal infrastructure to prevent re-identification and to protect privacy.

Rapid, lower-cost WGS is a promising research tool with unproven clinical utility, except in a small set of very specific situations. The journey from bench to bedside is one we should travel with care. Caution is warranted because we must reconcile diverse tensions—the commercial appetite for market growth versus the need for prudent health care expenditure, the research community's enthusiasm for genomic science versus professional, and public skepticism about personalized medicine. Due diligence should attend to the many competing demands on health care expenditure and biomedical R&D, to the ambiguous effects of new technologies, and to our well-justified ambivalence about the utility of an over-abundance of clinical data in the absence of evidence to establish actual clinical value. The scale and pace of adoption of this powerful new technology should be driven by clinical need, clinical evidence, and a commitment to put patients at the centre of health care policy.

## References

[1] Pasche B, Absher D (2011) Whole-genome sequencing: a step closer to personalized medicine. JAMA 305: 1596–1597. 21505140 10.1001/jama.2011.484

[2] Milner LC, Garrison NA, Magnus D, Cho M (2013) Genomics in the clinic: assessing the landscape of next-generation sequencing in diagnostic laboratories. In: American College of Medical Genetics Annual Meeting. Available: http://ww2.aievolution.com/acm1301/index.cfm?do=abs.viewAbs&abs=1663

[3] Hartocollis A (2013 April 21) Cancer centers racing to map patients' genes. The New York Times Available: http://www.nytimes.com/2013/04/22/health/patients-genes-seen-as-future-of-cancer-care.html.

[4] Leese S (2013 April 30) US institutions in genomic ‘arms race’. PHG Foundation Available: http://www.phgfoundation.org/news/13889/.

[5] Kilpivaara O, Aaltonen LA (2013) Diagnostic cancer genome sequencing and the contribution of germline variants. Science 339: 1559–1562. 23539595 10.1126/science.1233899

[6] Yu Y, Wu BL, Wu J, Shen Y (2012) Exome and whole-genome sequencing as clinical tests: a transformative practice in molecular diagnostics. Clin Chem 58: 1507–1509. 23065473 10.1373/clinchem.2012.193128

[7] Markoff J (2012 March 7) Cost of gene sequencing falls, raising hopes for medical advances. The New York Times Available: http://www.nytimes.com/2012/03/08/technology/cost-of-gene-sequencing-falls-raising-hopes-for-medical-advances.html.

[8] Lin JS, Thompson M, Goddard KAB, Piper MA, Heneghan C, et al (2012) Evaluating genomic tests from bench to bedside: a practical framework. BMC Med Inform Decis Mak 12: 117. 23078403 10.1186/1472-6947-12-117 PMC3538070

[9] Marshall E (2012 May 21) Prostate cancer test gets a failing grade. Science Insider Available: http://news.sciencemag.org/2012/05/prostate-cancer-test-gets-failing-grade.

[10] Ioannidis JPA (2013) Biomarker failures. Clin Chem 59: 202–204. 22997282 10.1373/clinchem.2012.185801

[11] Kolata G (2012 July 7) In treatment for leukemia, glimpses of the future. The New York Times Available: http://www.nytimes.com/2012/07/08/health/in-gene-sequencing-treatment-for-leukemia-glimpses-of-the-future.html.m.

[12] Dolan KA (2013 June 5) Marc Benioff, Mark Zuckerberg and healthcare movers gather at UC San Francisco summit. Forbes Available: http://www.forbes.com/sites/kerryadolan/2013/05/06/marc-benioff-mark-zuckerberg-and-healthcare-movers-gather-at-uc-san-francisco-summit/.

[13] Hayden EC (2011 June 15) Genome study solves twins' mystery condition. Nature doi:10.1038/news.2011.368. Available: http://www.nature.com/news/2011/110615/full/news.2011.368.html

[14] Asadi NB (2013 January 27) The personalized medicine revolution is almost here. Venture Beat Available: http://venturebeat.com/2013/01/27/the-personalized-medicine-revolution-is-almost-here/.

[15] Kimmelman J (2009) Gene transfer and the ethics of first-in-human experiments: lost in translation. New York: Cambridge University Press. 218 p.

[16] Hedgecoe A (2004) The politics of personalized medicine: pharmacogenetics in the clinic. New York: Cambridge University Press. 208 p.

[17] Hall WD, Mathews R, Morley KI (2010) Being more realistic about the public health impact of genomic medicine. PLoS Med 7 doi:10.1371/journal.pmed.1000347 10.1371/journal.pmed.1000347 PMC2953533 20967240

[18] Vera-Badillo FE, Shapiro R, Ocana A, Amir E, Tannock IF (2013) Bias in reporting of end points of efficacy and toxicity in randomized, clinical trials for women with breast cancer. Ann Oncol 24: 1238–1244. 23303339 10.1093/annonc/mds636

[19] Stenne R, Hurlimann T, Godard B (2012) Are research papers reporting results from nutrigenetics clinical research a potential source of biohype? Account Res 19: 285–307. 23009269 10.1080/08989621.2012.718681

[20] Kimmelman J (2012 December 19) In search of genomic incentives. The Globe and Mail Available: http://www.theglobeandmail.com/news/national/time-to-lead/in-search-of-genomic-incentives/article6534106/.

[21] Rinaldi A (2012) To hype, or not to(o) hype. EMBO reports 13: 303–307. 22422003 10.1038/embor.2012.39 PMC3321168

[22] Young NS, Ioannidis JPA, Al-Ubaydli O (2008) Why current publication practices may distort science. PLoS Med 5: e201 doi:10.1371/journal.pmed.0050201 18844432 10.1371/journal.pmed.0050201 PMC2561077

[23] Yavchitz A, Boutron I, Bafeta A, Marroun I, Charles P, et al (2012) Misrepresentation of randomized controlled trials in press releases and news coverage: a cohort study. PLoS Med 9: e1001308 doi:10.1371/journal.pmed.1001308 22984354 10.1371/journal.pmed.1001308 PMC3439420

[24] Fujimura JH (1988) The molecular biological bandwagon in cancer research: where social worlds meet. Soc Probl 35: 261–283.

[25] Caulfield T, Condit C (2012) Science and the sources of hype. Public Health Genomics 15: 209–217. 22488464 10.1159/000336533

[26] Office of New York State Governor Andrew M. Cuomo (2013 January 30) Governor Cuomo announces continued growth at Roswell Park Cancer Institute with regional economic development council funding. Roswell Park Cancer Institute Available: http://www.roswellpark.org/media/news/governor-cuomo-announces-continued-growth-roswell-park-cancer-institute-regional-econom-1.

[27] Wetterstrand KA (2013) DNA sequencing costs: data from the NHGRI Genome Sequencing Program (GSP). National Human Genome Research Institute Available: https://www.genome.gov/sequencingcosts/.

[28] Davies K (2010 October 1) The $1,000,000 genome interpretation. Bio-IT World Available: http://www.bio-itworld.com/2010/10/01/interpretation.html.

[29] Miller FA, Hurley J, Morgan S, Goeree R, Collins P, et al.. (2002) Predictive genetic tests and health care costs: a policy framework and illustrative estimates. McMaster University Centre for Health Economics and Policy Analysis research working paper 02-03. Hamilton (Ontario): McMaster University Centre for Health Economics and Policy Analysis.

[30] Fang J (2013 February 28) Soon, everybody will be sequenced. SmartPlanet Available: http://www.smartplanet.com/blog/bulletin/soon-everybody-will-be-sequenced/13856.

[31] Strickland E (2013 March 8) Should healthy people get their genomes sequenced? Discover Magazine Available: http://blogs.discovermagazine.com/crux/2013/03/08/should-healthy-people-get-their-genomes-sequenced.

[32] Bainbridge MN, Wiszniewski W, Murdock DR, Friedman J, Gonzaga-Jauregui C, et al (2011) Whole-genome sequencing for optimized patient management. Sci Transl Med 3: 87re3. 10.1126/scitranslmed.3002243 PMC3314311 21677200

[33] Mardis ER, Wilson RK (2009) Cancer genome sequencing: a review. Hum Mol Genet 18: R163–R168. 19808792 10.1093/hmg/ddp396 PMC2758710

[34] Evans JP, Meslin EM, Marteau TM, Caulfield T (2011) Deflating the genomic bubble. Science 331: 861–862. 21330519 10.1126/science.1198039

[35] Knottnerus JA, editor (2002) The evidence base of clinical diagnosis. London: BMJ Books. 226 p.

[36] Lee SSJ (2013) American DNA: the politics of potentiality in a genomic age. Curr Anthropol 54: S77–S86.

[37] Juengst ET, Settersten RA, Fishman JR, McGowan ML (2012) After the revolution? Ethical and social challenges in "personalized genomic medicine". Per Med 9: 129–139. 10.2217/pme.12.37 PMC3646379 23662108

[38] Prainsack B, Reardon J, Hindmarsh R, Gottweis H, Naue U, et al (2008) Personal genomes: Misdirected precaution. Nature 456: 34–35. 18987720 10.1038/456034a

[39] Juengst ET, Flatt MA, Settersten RA (2012) Personalized genomic medicine and the rhetoric of empowerment. Hastings Center Report 42: 34–40. 22976411 10.1002/hast.65 PMC3641662

[40] Marteau TM, French DP, Griffin SJ, Prevost AT, Sutton S, et al (2010) Effects of communicating DNA-based disease risk estimates on risk-reducing behaviours. Cochrane Database Syst Rev CD007275. 20927756 10.1002/14651858.CD007275.pub2 PMC12853416

[41] Collins RE, Wright AJ, Marteau TM (2011) Impact of communicating personalized genetic risk information on perceived control over the risk: a systematic review. Genet Med 13: 273–277. 20921892 10.1097/GIM.0b013e3181f710ca

[42] Bloss CS, Schork NJ, Topol EJ (2011) Effect of direct-to-consumer genomewide profiling to assess disease risk. N Engl J Med 364: 524–534. 21226570 10.1056/NEJMoa1011893 PMC3786730

[43] Caulfield T, Chandrasekharan S, Joly Y, Cook-Deegan R (2013) Harm, hype and evidence: ELSI research and policy guidance. Genome Med 5: 21. 23534337 10.1186/gm425 PMC3707044

[44] Marteau TM, Hollands GJ, Fletcher PC (2012) Changing human behavior to prevent disease: the importance of targeting automatic processes. Science 337: 1492–1495. 22997327 10.1126/science.1226918

[45] Wright CF, Gregory-Jones S (2010) Size of the direct-to-consumer genomic testing market. Genet Med 12: 594. 20697288 10.1097/GIM.0b013e3181ead743

[46] Evans JP, Berg JS (2012 December 1) The value of your genome. The Scientist Available: http://www.the-scientist.com/?articles.view/articleNo/33365/title/The-Value-of-Your-Genome/.

[47] Evans JP, Berg JS, Olshan AF, Magnuson T, Rimer BK (2013) We screen newborns, don't we?: Realizing the promise of public health genomics. Genet Med 15: 332–334. 23470837 10.1038/gim.2013.11 PMC4789099

[48] Collins FS (2010) The language of life: DNA and the revolution in personalized medicine. New York: HarperCollins Publishers. 368 p.

[49] Hagenkord J (2012 May 17) Empowering patients in the age of genomic medicine. Complete genomics Available: http://www.completegenomics.com/blog/Empowering-Patients-in-the-Age-of-Genomic-Medicine-151880905.html.

[50] Duke Center for Personalized Medicine About Us. Duke Personalized Medicine Available: http://www.dukepersonalizedmedicine.org/about_us/.

[51] Personalized Medicine Coalition (2009) About the Personalized Medicine Coalition (PMC). Personalized Medicine Coalition Available: http://www.personalizedmedicinecoalition.org/about.

[52] Teng K Message from the Director. Cleveland Clinic Center for Personalized Genetic Healthcare Available: http://my.clevelandclinic.org/cph/about-us/message-chair.aspx.

[53] Holman CM (2012) Debunking the myth that whole-genome sequencing infringes thousands of gene patents. Nat Biotechnol 30: 240–244. 22398617 10.1038/nbt.2146

[54] Nicholson Price II W (2012) Unblocked future: Why gene patents won't hinder whole-genome sequencing and personalized medicine. Cardozo Law Rev 33: 1–37.

[55] Nuffield Council on Bioethics (2002) The ethics of patenting DNA: a discussion paper. London: Nuffield Council on Bioethics.

[56] Report of the Secretary's Advisory Committee on Genetics, Health, and Society (2010) Gene patents and licensing practices and their impact on patient access to genetic tests. Secretary's Advisory Committee on Genetics, Health, and Society Available: http://oba.od.nih.gov/oba/sacghs/reports/sacghs_patents_report_2010.pdf.

[57] Kesselheim Aaron S, Cook-Deegan RM, Winickoff DE, Mello MM (2013) Gene patenting – supreme court finally speaks. New England J Med 369: 869–875. 23841703 10.1056/NEJMhle1308199 PMC3777541

[58] Cook-Deegan R, Conley JM, Evans JP, Vorhaus D (2012) The next controversy in genetic testing: clinical data as trade secrets? Eur J Hum Genet 1–4. 23150081 10.1038/ejhg.2012.217 PMC3658186

[59] Kolata G (2013 April 12) DNA project aims to make public a company's data on cancer genes. The New York Times Available: http://www.nytimes.com/2013/04/13/health/dna-project-aims-to-make-companys-data-public.html?pagewanted=all.

[60] Reg4All (2013) Registry for All. Available: http://reg4all.org/.

